# Using Multimodal Assessments to Capture Personalized Contexts of College Student Well-being in 2020: Case Study

**DOI:** 10.2196/26186

**Published:** 2021-05-11

**Authors:** Jocelyn Lai, Amir Rahmani, Asal Yunusova, Alexander P Rivera, Sina Labbaf, Sirui Hu, Nikil Dutt, Ramesh Jain, Jessica L Borelli

**Affiliations:** 1 UCI THRIVE Lab Department of Psychological Science University of California, Irvine Irvine, CA United States; 2 Department of Computer Science University of California, Irvine Irvine, CA United States; 3 School of Nursing University of California, Irvine Irvine, CA United States; 4 Institute for Future Health University of California, Irvine Irvine, CA United States; 5 Department of Statistics University of California, Irvine Irvine, CA United States; 6 Department of Economics University of California, Irvine Irvine, CA United States; 7 Department of Electrical Engineering and Computer Science University of California, Irvine Irvine, CA United States; 8 Department of Cognitive Science Irvine, CA United States

**Keywords:** COVID-19, emerging adulthood, multimodal personal chronicles, case study, wearable internet of things, individualized mHealth, college students, mental health

## Abstract

**Background:**

The year 2020 has been challenging for many, particularly for young adults who have been adversely affected by the COVID-19 pandemic. Emerging adulthood is a developmental phase with significant changes in the patterns of daily living; it is a risky phase for the onset of major mental illness. College students during the pandemic face significant risk, potentially losing several protective factors (eg, housing, routine, social support, job, and financial security) that are stabilizing for mental health and physical well-being. Individualized multiple assessments of mental health, referred to as *multimodal personal chronicles*, present an opportunity to examine indicators of health in an ongoing and personalized way using mobile sensing devices and wearable internet of things.

**Objective:**

To assess the feasibility and provide an in-depth examination of the impact of the COVID-19 pandemic on college students through multimodal personal chronicles, we present a case study of an individual monitored using a longitudinal subjective and objective assessment approach over a 9-month period throughout 2020, spanning the prepandemic period of January through September.

**Methods:**

The individual, referred to as Lee, completed psychological assessments measuring depression, anxiety, and loneliness across 4 time points in January, April, June, and September. We used the data emerging from the multimodal personal chronicles (ie, heart rate, sleep, physical activity, affect, behaviors) in relation to psychological assessments to understand patterns that help to explicate changes in the individual’s psychological well-being across the pandemic.

**Results:**

Over the course of the pandemic, Lee’s depression severity was highest in April, shortly after shelter-in-place orders were mandated. His depression severity remained mildly severe throughout the rest of the months. Associations in positive and negative affect, physiology, sleep, and physical activity patterns varied across time periods. Lee’s positive affect and negative affect were positively correlated in April (r=0.53, *P*=.04) whereas they were negatively correlated in September (r=–0.57, *P=*.03). Only in the month of January was sleep negatively associated with negative affect (r=–0.58, *P=*.03) and diurnal beats per minute (r=–0.54, *P*=.04), and then positively associated with heart rate variability (resting root mean square of successive differences between normal heartbeats) (r=0.54, *P=*.04). When looking at his available contextual data, Lee noted certain situations as supportive coping factors and other situations as potential stressors.

**Conclusions:**

We observed more pandemic concerns in April and noticed other contextual events relating to this individual’s well-being, reflecting how college students continue to experience life events during the pandemic. The rich monitoring data alongside contextual data may be beneficial for clinicians to understand client experiences and offer personalized treatment plans. We discuss benefits as well as future directions of this system, and the conclusions we can draw regarding the links between the COVID-19 pandemic and college student mental health.

## Introduction

### Emerging Adulthood and College

Young adults between the ages of 18-25 years report greater feelings of loneliness than other age groups [1]. Emerging adulthood is a developmental period marked by tremendous growth and opportunity but also by significant risk [2,3]. It is during this phase that the majority of major disease processes have their onset [4-7]; this is also a time when emerging adults develop habits for adaptive ways of managing stress and help-seeking behaviors [8]. The health habits formed during emerging adulthood have the potential to influence well-being throughout the lifespan [9], leading to the argument that this is a phase characterized by both risk and opportunity. The high level of plasticity during this phase can be attributed to various factors, including the lack of structure and increased self-directed activity [10,11], shifting roles and responsibilities [12], and relatively less institutional structure to support development [13]. A large proportion of these individuals pursue higher education. The college experience is a unique time in which this age range typically faces a myriad new experiences and opportunities for growth, development, and risk. As such, college students are an important subgroup of emerging adults that are worthy of investigation.

College students are a diverse cross-section of the population [14], coexisting in environments with less structure imposed by parents [15]. They are likely to be exposed to high doses of peer influence [16-18], less regulation in sleep and wake cycles [19,20], and less adult oversight of risk-taking behaviors [21]. Alongside developmental shifts, the college experience in combination with academic, career, and socioeconomic pressures may create a perfect storm for the onset of mental health problems. However, stability in academic, career, and financial livelihood during this time characterized by the exploration of one’s self-identity—in the form of active college student life, social support, and financial aid—are considered protective factors that sustain well-being [22]. Studies suggest that college students with higher levels of stress often have poor mental health outcomes [23]. Furthermore, they are more likely to engage in maladaptive behaviors such as substance use [24] and less likely to engage in health-promoting and adaptive help-seeking behaviors like accessing mental health services [25], asking professors for help [26], exercising [27], healthy eating [28], and practicing sleep hygiene [29]. One way this has been addressed is through technology. Emerging adults are also the largest subgroup of the population to rely on technology [30,31]. Over 30% of teens and young adults in the United States report using digital platforms as a method for health tracking and monitoring [32]. Together, these factors provide support for the real-world case and importance of stress management and mental health promotion among young adults.

### The Year 2020 and the COVID-19 Pandemic

The year 2020 has largely been defined by the COVID-19 pandemic, which has disrupted the daily lives of people across the world. All of the aforementioned health-related concerns for college student mental health have been exacerbated by the COVID-19 pandemic [33,34]; universities across the states have converted into remote learning centers, sending college students home, disrupting their previous routines and daily rhythms, upending job prospects and the economy, and increasing uncertainty and anxiety across the United States [35-39]. Altogether, a tremendous shift has occurred from the excitement and opportunities of collegiate life to turbulence, trauma, and stress, relating to increased rates of mental health problems in this population [40-43]. The uncertainty with the ongoing pandemic renders many of the stabilizing factors in the college experience even harder to attain; therefore, there may be added stressors in maintaining social support networks and uncertainties with financial support for current college students, as well as worries about the quality of one’s education and career prospects for students close to finishing their degree. In addition to the pandemic, sociopolitical unrest and a series of other potential stressors have arisen throughout 2020 (eg, the murder of George Floyd, the Black Lives Matter movement) that may have been of concern to students [44,45]. Because the state of the environment is intricately associated with daily life and well-being [46,47], it is important to contextualize the nuanced experiences of mental health with relevant immediate and larger, externally occurring events.

### Multimodal Assessments and Personalized Approaches of Well-being

Examining student wellness in the context of their experiences may help to inform programming and intervention-based efforts to maintain student mental health, a key priority of campus wellness. From a biopsychosocial framework, wellness not only embodies internal functioning but also the social and external environment [48,49]. For each individual, the interaction of their own genetic and biological systems combines with their psychological and social systems [48,49]; the resultant psychopathology may differ. Thus, assessing numerous systems and factors are necessary to fully capture an individual’s mental health trajectory.

Precision medicine and idiographic approaches to health have gained traction to address the heterogeneity of symptomatology [50,51]. With advancements in technology, researchers have utilized and are continuing to refine mobile, passive sensing to best capture lived experiences and improve ecological validity at the individual level [52]. Furthermore, these technological and methodological advancements have permitted researchers to pursue individualized approaches in examining mental health and informing personalized treatment [51,53-55]. These methods allow for continuous measurement of health markers such as physiology, sleep, and physical activity, contributing to rich and large amounts of data at the individual level. Use of wearable and mobile devices to capture data in daily life, known as the internet of things (IoT) and wearable IoT (WIoT) can help process and synthesize multiple forms of assessments (ie, geographical, physiological, behavioral, subjective, and contextual data) that may aid a health professional in offering guidance to an individual seeking mental health treatment [56,57]. Therefore, understanding well-being and the contexts relevant to the trajectory of an individual’s mental health may benefit from a whole-systems approach using WIoT and ecological momentary assessments. In combination with active sensing, multimodal personal chronicles, as we define it, incorporate these multiple forms of assessment (eg, physiology, subjective experiences, contexts) as a way to capture unique, personal contexts of an individual’s daily lived experiences. Young adults are avid users of technology, being more dependent on their smartphones and using social media compared to adults ≥30 years of age [30,31]. As such, we note that young adults, and college students in particular, may be an ideal population to examine the feasibility of the multimodal personal chronicles system as a method for examining individualized approaches.

### This Study

Given the novelty of the COVID-19 pandemic, we reasoned that the use of a case study was an important step in the scientific investigation of this phenomenon, one that would highlight the potential of the multimodal personal chronicles*.* The purpose of this case study is to provide a nuanced view of one individual’s subjective experience and objective bodily responses during a historic and unprecedented event, while underscoring the potential of a research methodology for assessing the heterogeneity of well-being among college students. Case studies are effective methods for exploring contextual phenomenon within a single or small unit (eg, N=1 or small sample) in a given spatial and temporal context; they are often used when examining a newly emerging or poorly understood phenomenon [58,59]. These approaches may be advantageous in enhancing our understanding of the pandemic experience and its relevance to college student well-being beyond that of just self-report. Furthermore, case studies allow researchers to assess the feasibility of novel approaches on a small scale [59]. Such approaches are particularly useful at the outset of a historical phenomenon or the beginning of a scientific investigation, when the accumulation of knowledge is in its infancy and a more fine-grained analysis may provide important information that could be lost if compressed into a study involving many individuals.

Our recent work [55] has demonstrated the feasibility of estimating stress levels by monitoring physiological signs of sympathetic nervous system activity (eg, heart rate, heart rate variability, respiration rate, galvanic skin response, etc). However, this method of objective monitoring only captures changes in internal states and not contextual factors—such as mental activity and social interactions—that are critical for the diagnosis, treatment, and prevention of mental health problems that may follow from the long-term effects of stress. There is a fundamental need to conduct studies that not only assess individual subjective and objective physiological reporting of health but also capture higher-level life events and contextual information to enable root cause analysis for treatment and prevention. Our purpose is to thus incorporate these multimodal assessments together in studies examining contexts. As prior studies have documented evidence of the negative associations of the pandemic among college students [33,34,40-43], capturing daily life during the pandemic, shelter-in-place orders, social distancing, and remote learning among college students may help to better understand the individualized increased risks for student mental health. In turn, this may ultimately further expand our understanding of the pandemic experience and its association with college student well-being. We present a case study of one participant from our larger pilot study to illustrate both (1) the potential and feasibility of a multimodal personal chronicles approach toward well-being and (2) the fine-grained experiences of one individual college student during the pandemic. Thus, our research questions are as follows:

1. How does a multimodal (passive and active) sensing approach via multimodal personal chronicles capture individualized and contextual experiences of well-being?
2. What is the experience of daily living for a college student in the context of the COVID-19 pandemic?

## Methods

### Procedure

We discuss the procedures for the larger study in which our case study is derived from. In January 2020, we began an Institutional Review Board (IRB)–approved (#2019-5153) investigation to pilot and examine the utility of this multimodal personal chronicles system in understanding changes in mental health symptoms (eg, depression, anxiety) and general psychological distress (eg, loneliness, negative emotion) over time among emerging adults in college. Participants completed a comprehensive psychological battery at study intake (eg, depression, anxiety, loneliness) and used devices within the multimodal system (ie, Oura ring, Samsung Gear Sport, ecological momentary assessments, and Personicle [60-62]) that assessed physiology, sleep, physical activity, step count, and affect. They then received another comprehensive battery 3 months later in April. Originally, we intended to end the pilot study after 3 months; however, concurrent to our original cohort’s 3-month participation period, the COVID-19 pandemic worsened in the United States and elsewhere. This pandemic had a profound impact on the livelihood of individuals across the world [63]; this prompted our investigative team to feel that it was important to capture the lived experiences of college students as it was occurring and to examine the association between the pandemic and student well-being from an intensive, longitudinal approach. At the onset of COVID-19 and the shutdown associated with this global pandemic, we had enrolled 13 college students into our immersive pilot; however, one of those participants was discontinued and two voluntarily withdrew early, resulting in a total sample size of 10. As a result, we extended our study, continuing participants’ data collection and incorporating in-depth psychological assessments once at 2 months (June) following the original study duration and then at 3 months (September) thereafter. Participants enrolled had the option to continue or end the study as planned.

### Measures

#### Psychological Battery

**Figure 1:**
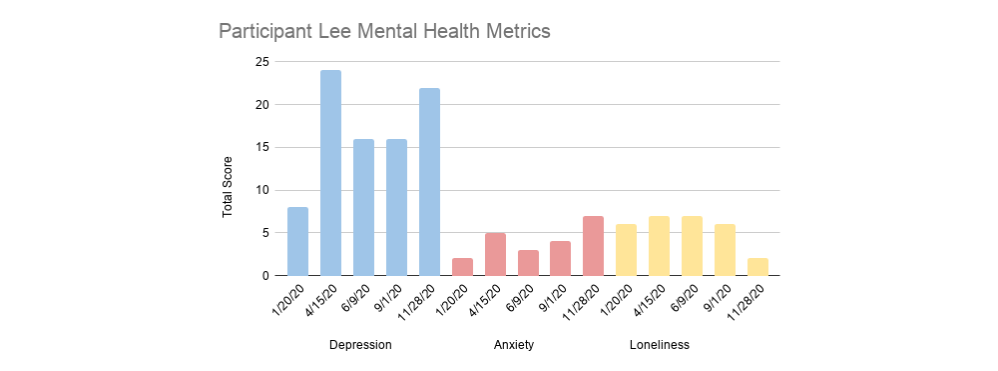
Lee’s depression, anxiety, and loneliness scores at 4 different time points in the study. Note: the sum of anxiety scores is reported rather than the means to show score distribution over time.

The battery of psychological and well-being–related questionnaires were administered at baseline in person and during subsequent follow-up assessments (see Figure 1 for a timeline) as an online survey link.

#### Depression

Participants completed the Beck Depression Index II (BDI-II) [64] to assess depressive symptoms. This is a 21-item, well-validated measure rated on a 4-point scale (0-3; α=.90-.91) [65,66] that allows individuals to indicate to what degree they are experiencing a feeling, for example, “I do not feel sad” (0) to “I am so sad or unhappy that I can’t stand it” (3) with the exception of 2 items using a 6-point scale to reflect different degrees of behavioral changes. Scores are calculated as a total, which indicates the range in depression from mild (14-19) to severe (29-63) [65]. If participants scored above 24 and/or endorsed suicidality at intake or any follow-up assessments, the principal investigator (author JLB, a licensed psychologist) reached out to these individuals to determine symptom severity and if the participant should continue with or be withdrawn from the study. These individuals were then provided additional mental health resources.

#### Anxiety

The Brief Symptom Inventory (BSI) [67], a 6-item anxiety subscale, was used to measure anxiety. The BSI is a commonly used measure that assesses a wide array of psychological symptom dimensions, including depression, somatization, and anxiety on a 5-point Likert-type scale (0=not at all, 4=extremely; α=.81-.86) [68,69]. Anxiety scores are typically calculated as a mean of all items (eg, “During the past 7 days how often were you distressed by nervousness or shakiness inside?”); for our purposes, we used the sum of all items to observe the distribution of participants’ anxiety score over a period of time alongside other psychological constructs of interest. A higher score indicates greater anxiety. Normative ratings among nonpsychiatric patients average around 0.35 (SD 0.45) [69].

#### Loneliness

Participants were administered the UCLA (University of California, Los Angeles) 3-item Loneliness Scale, a frequently used measure of perceived loneliness (eg, “How often do you feel you are left out?”; α=.72) [70]. The UCLA Loneliness Scale has been used widely in the literature to examine how loneliness relates to psychological and physiological outcomes (eg, mood, depression) [70]. Items are totaled and rated on a 3-point Likert-type scale (1=hardly ever, 3=often), where higher scores reflect greater perceived loneliness.

#### Multimodal Personal Chronicles

As part of the intensive active and passive data sensing, over the course of the study, we tracked participants’ emotional states, physiological patterns, and behavioral habits through WIoT devices and ecological momentary assessments.

##### Physiology, Sleep, and Behavioral Patterns

Participants were instructed to wear 2 devices (Oura ring and Samsung Gear Sport smartwatch) as well as download the corresponding Oura and Samsung Android mobile apps to measure and store their physiology, sleep, and behavioral patterns. The Oura ring [71] measures a wide array of personal variables across numerous categories, including heart rate, heart rate variability, respiration rate, skin temperature, sleep, and activity level (majority of these are monitored only overnight). For our investigation, we assessed sleep quality using the Oura ring’s personalized sleep score that uses a combination of individual body metrics (ie, height, weight, age, and gender) with a validated system that detects time spent in different stages of sleep [60,61]. The Samsung smartwatch tracks several user activities and physiological variables 24/7 (for more details on how these devices were used for our study, see Yunusova et al [72]). We were able to use the watch to assess participants’ heart rate variability (root mean square of successive differences between normal heartbeats [RMSSD]) and beats per minute (BPM) during diurnal periods (RMSSD and day BPM), and during the entire 24-hour period (RMSSD and resting BPM).

Lastly, the Personicle app [62] was downloaded onto each participant’s phone to observe behavioral trends. Personicle tracks users’ changes in location through the Google GPS application programming interface. For the purpose of this study, the participants’ step count was assessed through this app.

##### Ecological Momentary Assessments of Affect and Context

Participants completed daily assessments of positive and negative affect using the Positive and Negative Affect Schedule (PANAS), a widely used scale that has high internal consistency, validity, and reliability (for positive affect, α=.85; for negative affect, α=.91) [73]. This scale is highly correlated with measures of psychopathology and distress such as depression and anxiety [74,75]. In clinical samples, the PANAS scale can differentiate between anxiety and depression among individuals, with positive affect being highly correlated with depression and negative affect being highly correlated with both depression and anxiety [76,77]. Example items of positive affect items include “excited” and “inspired” while negative affect include items such as “afraid” and “nervous,” all rated on a sliding scale from “very slightly” (0) to “extremely” (100), where higher scores reflect higher positive or negative affect. This was sent as a survey prompt to participants’ mobile phone using a separately downloaded app that was designed specifically for the survey to be taken on, and was completed once a day between the hours of 8 pm and 2 am. Additionally, participants completed a weekly “feel-in” every Sunday afternoon between the hours of 8 am and 3 pm, where they were instructed to answer an open-ended question about the quality of their week (ie, “Please write about your high points and low points this week. Please try to be as detailed as possible”).

### Data Analytic Plan

We followed participants from January through September, in which each individual completed a total of 4 battery assessments of psychological well-being (across roughly 3-month time frames—January, April, June, and September) in addition to intensive longitudinal assessments of heart rate, heart rate variability measures, respiration rate, skin temperature, sleep quality, step count, and affect throughout the study period. We focused on heart rate (BPM), heart rate variability (RMSSD), sleep quality, step count, and affect for this case study.

For our data analytic plan, we will use a descriptive approach to illustrate the data over the course of 9 months, examining any changes in participants’ depression, anxiety, and loneliness ratings at each of the 4 assessments as well as their BPM, RMSSD, sleep quality, step count, and positive and negative affect aggregated across the 2-week period after completion of the psychological assessment. Then, we will run correlations to examine associations between the aggregated physiological, sleep, behavioral, and affect measures at each time point. Doing so offers a broader perspective of how these multimodal assessments relate to one another and how they may differ over the course of the pandemic.

We will then narrow down to explore 2-week periods of data to better understand contextual factors potentially relevant to the fluctuations in their emotional and physiological patterns over the 2-week period (ie, data points for each day for 14 days). Focusing on the occurrences at 4 different, 2-week time points may further help us observe relationships across multimodal measures at the daily level rather than aggregates across months. We note that the adjustments made to the study in response to the pandemic were approved by the IRB in April, thus limiting the available self-reported contextual data we can examine. Therefore, while we present the 2-week data for each of the 4 time points, we only describe in detail the contextual data for the periods of June and September. Finally, we will offer clinical recommendations based on the data derived from the multimodal personal chronicles as potential suggestions for the single subject this case study focuses on.

## Results

For the purposes of this case study, we focused on the results of one individual. Like many other participants in our study, this individual exhibited a decline in mental health as the pandemic-enforced period of social isolation extended. In order to preserve the confidentiality of this participant, we have modified details regarding this participant’s case.

**Figure 2:**
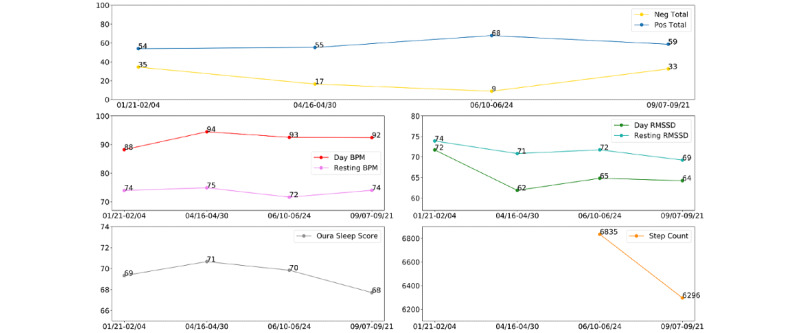
Lee’s multimodal assessments at the 4 different assessment time points in the study. Note: we did not collect data on step count through the mobile phone app until May, thus limiting our interpretation and examination of step count during the January and April periods. Neg: negative, pos: positive, BPM: beats per minute, RMSSD: root mean square of successive differences between normal heartbeats.

“Lee,” as we shall refer to this individual, is an Asian-American male student in the middle of his sophomore year of college. We report Lee’s pandemic narrative in chronological order since the start of his participation in the study in January through to September. Lee began the study with mental health symptoms that were low and not within the clinically significant range. For instance, his depression score at intake was 8, below the cut-off for mild depression (see Figure 1 for levels of depression, anxiety, and loneliness across each assessment time point). Figure 2 visually displays his 2-week aggregate score for all multimodal measures at each time point. Over the course of the pandemic, we observed that Lee reported relatively low levels of negative affect compared to his levels of positive affect, despite his higher depressive scores in April through September.

By the second time point in April, compared to his baseline assessment in January, his depression score had increased to 24—well within the moderate depression range. His anxiety and perceived feelings of loneliness also slightly increased (Figure 1). Because Lee had shown such an increase in his depression, roughly 3 times his initial levels in January, author JLB conducted a phone screening to assess his mental health. When JLB spoke to Lee, she learned that since the onset of COVID-19 toward the end of March, Lee had experienced more stress—a family member of his had experienced a significant stressor due to COVID-19, which in turn also impacted him. Lee’s mother had been living abroad in [country] as a visiting professor and was unable to return home before travel restrictions were imposed, resulting in her being unable to leave [country] for a period of months. This created a great deal of anxiety for everyone in Lee’s family—having Lee’s mother be separated from the family, apart from everyone during a global crisis, with no certainty regarding when she would be able to return, was highly distressing. During this time, Lee moved back home to live with his family, disrupting the social support and routine he had established in his college environment. Lee reported that his mood was low but that he was not feeling suicidal. JLB provided him with referrals and informed him that he could reach out to her at any time should he need additional support. Consistent with the study protocol, the research team and JLB continued to track Lee’s mood, sleep, physiology, and physical activity throughout the study.

Since his assessment in April, Lee’s depression remained in the mildly severe range in June and September. His reported anxiety had lowered since, but we noted that his loneliness was consistent between the periods of April and September. From his wearable sensor and ecological momentary assessment data, we noted that there were fairly minimal fluctuations in his physiology and sleep. Although his BPM was highest in April, it was not largely different from his BPM at other time points. His sleep was lower in the January and September periods, which reflect the time of the start of the winter and fall quarter, possibly relating to these differences than the April (spring quarter) and June (close to summer) periods. We report correlations of the measures at each time point in Multimedia Appendix 1 (Tables S1-S4). Based on the correlational data, associations varied across time periods. For example, Lee’s positive and negative affect were positively correlated in April (eg, high positive and high negative affect; r=0.53, *P*=.04) whereas they were negatively correlated in September (eg, high positive and low negative affect; r=–0.57, *P*=.03). BPM and RMSSD were relatively correlated throughout each time point (Multimedia Appendix 1, Tables S1-S4). Only in the month of January was sleep negatively associated with negative affect (r=–0.58, *P*=.03) and day BPM (r=–0.54, *P*=.04), and then positively associated with resting RMSSD (r=0.54, *P*=.04).

**Figure 3:**
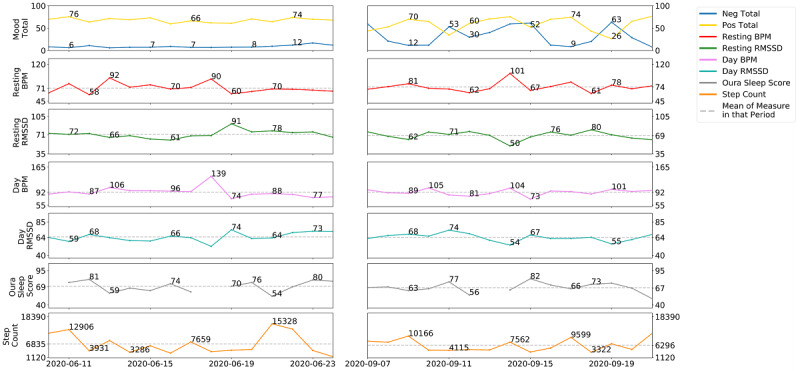
Raw numbers of all measures during the 2-week period on June 10-24 and September 7-21. Note: negative (neg) total, positive (pos) total, and Oura sleep score range between 0 and 100. Mood total: blue line signifies negative emotion and yellow line signifies positive emotion; resting beats per minute (BPM): sampled 15 minutes every 2 hours, including sleep; resting RMSSD (root mean square of successive differences between normal heartbeats): index of heart rate variability, sampled 15 minutes every 2 hours, including sleep; day (resting) BPM: sampled 15 minutes every 2 hours, not including sleep; day RMSSD: sampled 15 minutes every 2 hours, not including sleep; Oura sleep score: summary score of overall sleep quality, a weighted sum of sleep contributors, which combines sleep latency, onset, restfulness, rapid eye movement, deep sleep, timing, and efficiency; step counts: generated from the watch, total number of steps taken per day.

To further illustrate the potential of a personalized approach in helping to inform our understanding of college student mental health, we focused on the June and September 2-week time periods. Table 1 displays contextual data from Lee’s weekly responses, and Figure 3 highlights the trends in the multimodal assessments. More specifically, the figure displays the daily monitoring of Lee’s positive and negative affect, physiology, sleep, and physical activity. Based on Figure 3, in June, Lee experienced greater distinctions in his positive and negative affect, where he reported consistently higher levels of positive affect and consistently lower levels of negative affect. When observing Lee’s data during this time, his subjective weekly reporting of his high and low points (Table 1) suggested that he was socially engaged with peers (ie, hiking with friends, participating in the Black Lives Matter social movement with peers) and had a sense of purpose (eg, making social change) as well as accomplishment (eg, finishing a year of school).

**Table 1:** Lee’s weekly “feel-in” responses to weekly highs and lows across the June and September time frames.

Time frame and time stamp		Response	LIWC^a^ analysis
**Time 1 (June 10-24)**			Negative emotion word frequency=2.63; positive emotion word frequency=5.26
	Sun, Jun 7, 2020; 09:56:00	“Some high points this week are leading a talk on the Black Lives Matter movement with my [group of people] and [group of people] which was really successful. Another high point is completing three of my courses. A low point is having to listen to racist comments and people that don’t support the Black Lives Matter movement. Also, I still have to study for two more finals and a term paper”	
	Sun, Jun 14, 2020; 11:53:57	“High points include being done with [a year of school]. I went running with friends and had so much fun. I got to see the night sky and great views in [location redacted]. Low points include listening to my friends’ problems and trying to help them both through it. Another low point is hanging out with a friend and another person joined us who I don't like, so I didn’t really enjoy myself anymore.”	
	Sun, Jun 21, 2020; 12:45:24	“High points in my week are hiking and playing [game] with friends. I did not have any low points. This has been my best week in months”	
	Sun, Jun 28, 2020; 14:55:40	“Some high points include starting a [program] that will help me work on a project for the future as well as going to the beach with friends. Some low points include having to help friends through a difficult time, the large amount of work I have to take care of, and beginning to delay responsibilities and need to fix my schedule.”	
**Time 2 (September 7-21)**			Negative emotion word frequency=2.84; positive emotion word frequency=5.88
	Sun, Aug 30, 2020; 12:44:13	“The biggest high point of my week was being able to spend some more time with the [person] I like. We were able to eat dinner, talk, and walk on the boardwalk. It felt like nothing else really mattered and I let go of my responsibilities and things that I had to do. I also got to lead a group meant to hear [opinions] about career resources for the project that I am involved in. It felt good to hear from [group of people] and I want to be able to help them with their concerns. Low points this week was having to revise my [project] again because it didn’t meet certain expectations. It's irritating having to change it multiple times even though I already address the revisions that my [supervisor] requires.”	
	Sun, Sep 6, 2020; 14:16:27	“The high points in my week are my hikes with people. It feels good to hike and being active again since I cannot play games. I also was able to complete a project for a [group of people] that I hope gets approved by the [group of people]. These past few days have really sucked because I wasn’t able to work on my [project] and being stuck. It's been frustrating and I have a difficult time when I’m not able to do work and I’ve been having [sic] feelings of inadequacy. It's been hard but one of my best friends has been really helpful because they always reassure me and give me words of affirmation.”	
	Sun, Sep 13, 2020; 11:55:19	“This past week has had such ups and downs with so many emotions. I was sad to discover that my cousin, who was assaulted last year at [name of a different college campus], has been going through a difficult time and [they’ve] been experiencing triggers. I got angry with myself for not being able to physically support [them] last year when she needed it most. I was able to talk on the phone and text them now which was helpful but I wanted to be there in person to show that I'm there for [them]. Another low point is studying for an upcoming [a test]. The grade isn’t that important to me but I want to do good. A couple high points is having a virtual get together. It was great to hear their voices and see their smiles. I also got to spend a day with my friend and had a lot of fun. I also finished another draft of my [project] and that was giving me headaches all week.”	
	Sun, Sep 20, 2020; 16:10:00	“This week had it's [sic] highs and lows. A high is finishing the [test] which felt great. Another high is being accepted into [a program] but the low part of that is finalizing edits which has been really hard to get a start on. Another low is finding out my friends [relative] passed away in [country and helping [them] through it. It's been hard for [them], especially with [their] other [relative] passing away recently.”	

^a^LIWC: Linguistic Inquiry Word Count System.

In contrast, during September, Lee exhibited more erratic mood states, which we interpret to potentially be an indication of low well-being. Lee’s negative and positive affect show greater variations from day to day. Compared to when Lee’s positive affect was more distinct from his negative affect in June, his negative affect was nearly as high as his positive affect in September. Further, in September, he experienced days in which his negative affect was higher than his positive affect. Between the two time periods, there were relatively similar fluctuations in his physical activity, heart rate levels, heart rate variability (as indexed by the RMSSD score), sleep, and step count (Figure 3). He also had fewer social contact, as reflected in his weekly self-reported high and low points (Table 1). As an additional exploratory analysis, we analyzed the narrative responses of Lee during his weekly “feel-ins” using the Linguistic Inquiry Word Count System [78]; our findings revealed that Lee used more negative (*t*_1_=26.05, *P*=.02) than positive words (*t*_1_=17.97, *P*=.04) in September than in June, further reflecting greater variation in his affect.

## Discussion

### Principal Results

The use of a case study enabled us to closely examine the experience and daily life of Lee across distant time points within the past year. Lee’s depressive symptoms increased sharply from January to April, a time coinciding with the onset of the COVID-19 pandemic and supporting prior evidence for increased depressive symptoms and distress among young adults during this period [38,39,47,48]. When examining his data more closely at a 2-week period with his contextual responses, Lee reported greater negative affect and use of negative emotion words in the month of September than in June. As a rising college senior, Lee may have felt the winding down of the previous academic year in the month of June while feeling the build-up of an eventful start to the academic year in the month of September. Courses were transitioned remotely at the end of March due to COVID-19; while this may have resulted in increased stress and uncertainty in April, students may have felt some relief at the anticipation of summer, which heralds a reduction in academic stressors. With regard to September, students may have felt a rise in stress and uncertainty with what the upcoming school year will look like. To inquire about student concerns, one department at the university had conducted a series of undergraduate focus groups in the month of September. Students overall had expressed unease regarding the prolonged period of remote learning and wondered whether instructors would expect students to have adjusted and thus no longer offer flexibility in their courses in the upcoming quarter. Much like the students in the focus group, Lee may have felt increased stress surrounding the uncertainty of the upcoming school year, which could be associated with increases in his experiences of negative affect.

We are able to understand Lee’s unique pandemic experiences from this longitudinal assessment. Although his depression lowered from severe to mildly severe in the following months from April, Lee’s loneliness score remained consistent between the months of April through September, possibly relating to the continued social distancing and having to rely on alternative methods of keeping in contact with friends (eg, online chatting and hangouts). We also observed relatively higher levels of positive affect and lower levels of negative affect throughout Lee’s participation in the study. His negative affect was lowest in June, which is reflected in the 2-week period we examined more closely. While he may have experienced external events that affected his depressive symptoms (eg, his mother’s situation), there were potentially supportive factors (eg, social networks, meaningful work) that sustained his positive affect while maintaining lower negative affect during the pandemic. Thus, the meaning and significance of the multimodal data becomes most clear when aligned with the contextual data.

With regard to the pandemic, the approach allowed us to understand fluctuations in health and well-being that are linked to the unique experiences an individual may encounter during the pandemic. For example, the situation with Lee’s mother was a significant stressor for him, and this was only possible to document with the multiple assessments of depression and having this data accessible to a clinical psychologist to follow up with him regarding his well-being. Alternatively, we also recognize that there were some fluctuations that were relatively low (eg, physiology across the 4 time periods as well as in the 2-week period of June and September). This may also point to important coping and resiliency factors that allow individuals like Lee to navigate this stressful pandemic. With any kind of stressful event, Lee may be using his current resources and available coping strategies to self-regulate during the pandemic.

The case study highlights preliminary data from our larger ongoing study, which in turn enables researchers and clinicians to better understand the context and unique experiences of individuals and how these factors relate to well-being. The utility of multimodal personal chronicles for mental health may be clearest when it is used in conjunction with the collaborative care of a clinician. From a health navigation framework [79], health professionals can access this data, synthesize it with their current knowledge of the individual, and offer informed guidance and treatment recommendations [80]. Researchers may also consider using advanced modeling or network analyses [81,82] to examine this intensive, longitudinal approach as a way to understand the interconnection of biological, psychological, and social factors relevant to well-being.

### Recommendations

Access to such rich amounts of personalized data can help clinicians or therapists offer evidence-based treatments or recommendations. If the provider in the loop were to offer recommendations to Lee based on his pattern of behaviors, it would be to increase and maintain a moderate to ideal level of physical and interpersonal activity with his support networks. As noted, based on his weekly open-ended responses, many of his high points included spending time with friends or enjoying time outdoors doing either physical activity or enjoying nature with others. Interpersonal support and interactions were particularly relevant, and thus seeking strategies to ensure consistent social connectedness with others may be helpful for Lee in sustaining well-being. Given that public health recommendations of social distancing preclude in-person social interactions, Lee may wish to savor memories of times when he has enjoyed spending time with other people (eg, relational savoring [83]), which has been shown to increase positive emotion and reduce depression. Similarly, attending to and prolonging the positive emotions associated with one’s romantic partner has shown to have a positive impact on those in long-distance relationships [84], suggesting that this technique may also assist people during periods of social distancing. Furthermore, Lee noted being involved in purposeful work facilitating discussions relating to sociopolitical movements and supporting wellness efforts on campus. Creating meaning and finding a sense of purpose are linked to subjective well-being [85,86], and so continuing to find meaning in his work or savoring these feelings that coincide with the work he finds meaningful may also be particularly helpful in sustaining mental health. Additional recommendations could potentially include engaging in positive self-reflections, such as savoring interpersonal or achievement moments throughout the week. These reflections may also be helpful in the form of gratitude journaling or writing down 3 things they were grateful for that day. Lee’s low points were often linked to academic and interpersonal stressors; focusing on his small achievements can help Lee focus on his competency and ability to be resilient despite in-the-moment stressors. While each week may include life challenges, Lee can recall his own strengths and resiliency to stay motivated and overcome various stressors.

### Limitations

Limitations to our study include the fact that, as with many case studies, it is primarily exploratory in our understanding of a phenomenon in a specific space and time. As we are considering the feasibility of multimodal personal chronicles, we restrain from making causal claims regarding the experiences of Lee. Additionally, self-reported emotion data were only captured once a day toward the evening, which allows for recall bias. Recent studies are now suggesting that the PANAS scale is in need of improvement [87] while also pointing out that positive and negative affect are not completely independent dimensions [88]. Furthermore, due to a data collection error, we did not have weekly self-reported “feel-in” data available for Lee until approximately 1 month after the initial shelter-in-place order. Thus, we were only able to examine the contextual data alongside the active and passive sensing data for June onwards. Because we selected a 2-week period for the time points, there were limited data to examine when conducting statistical analyses. Thus, the case study may benefit from including more observations and a larger study including more participants. Once the full study is complete, we intend to use analytical techniques (eg, structural equation modeling), which will permit us to use a larger number of observations in our data to understand our participants’ mental health trajectories.

### Comparison With Prior Work and Future Directions

Emerging adults navigate a number of stressors during this developmental period; experiencing all of this while facing a global pandemic may exacerbate psychopathology or detriments to psychological well-being among college students. For example, students may have to juggle negative impacts of the pandemic on their family while also adjusting to online and remote learning. Previous studies examining student well-being during the pandemic have largely conducted single time-point assessments [38,45,46,48] or are limited to self-report assessments [39,47]; our study utilizes an intensive longitudinal case study to showcase how the year throughout the pandemic has not only affected student mental health but also potentially through indirect means like student livelihood and daily experiences. The study contributes to our understanding of how college students may cope with a global stressor, in that some may navigate stress similarly to how they cope with other life stressors. Indeed, much of the current literature on the pandemic has focused on adverse health outcomes, and so future investigations may consider coping and resiliency among individuals through this period.

The feasibility of multimodal personal chronicles to assess mental health may help to inform what types of interventions may be best for individuals. Future studies may consider assessing the feasibility of the multimodal personal chronicles in other populations (eg, clinical samples, across cultures) and incorporating interventions or guidance in the process of data collection as a way to examine how self-monitoring of health alongside guidance may then have trickle-down effects on improving health. In addition, this approach offers the potential for a system in which individuals can tweak their own health behaviors and see its associations with changes in their health trajectory [89,90]. Health professionals may also consider how different types of strategies may be beneficial at different times, depending on the context and experiences of the individual [84]. Indeed, a personalized approach grants flexibility in treatment and interventions in ways that address the dynamic nature of health and well-being.

### Conclusion

From our ongoing study, we present results from a case study examining 1 participant and how the trajectory of his well-being may be assessed through a holistic multimodal personal chronicles system that captures personalized experiences. Case studies have the benefit of presenting in-depth information regarding a single individual, enabling a fine-grained analysis of the circumstances, characteristics, and experiences for that person at a specific point in time. Future studies may consider the multimodal personal chronicles approach and how it may inform treatment and intervention to mitigate psychopathology and aid in maintaining well-being.

## Appendix

Multimedia Appendix 1 Correlation tables of the 2-week assessments for January, April, June, and September.

## Abbreviations

BDI-II: Beck Depression Index II
BPM: beats per minute
BSI: Brief Symptom Inventory
IoT: internet of things
IRB: Institutional Review Board
PANAS: Positive and Negative Affect Schedule
RMSSD: root mean square of successive differences between normal heartbeats
UCLA: University of California, Los Angeles
WIoT: wearable IoT
