# Supplementary material for: Using Multimodal Assessments to Capture Personalized Contexts of College Student Well-being in 2020: Case Study
Source: JMIR Form Res. 2021 May 11;5(5):e26186. doi: 10.2196/26186 (PMC8115397; doi:10.2196/26186)
Supplement: Multimedia Appendix 1 [file formative_v5i5e26186_app1.docx]

Multimedia Appendix 1: Correlation Tables of the 2-Week Assessments for January, April, June, and September

Table S1. Correlation between 2-weeks of daily assessment for the month of January

|  | **Positive Affect** | **Negative Affect** | **day_bpm** | **day_ rmssd** | **resting_bpm** | **resting_ rmssd** | **oura_sleep_score** |
| --- | --- | --- | --- | --- | --- | --- | --- |
| **Positive Affect** | 1 | -0.37 | -0.09 | 0.01 | -0.19 | -0.2 | 0.25 |
| **Negative Affect** |  | 1 | 0.13 | -0.02 | -0.14 | 0.06 | -0.58* |
| **day_bpm** |  |  | 1 | -0.85*** | 0.45 | -0.84*** | -0.54* |
| **day_rmssd** |  |  |  | 1 | -0.47 | 0.76*** | 0.51 |
| **resting_bpm** |  |  |  |  | 1 | -0.44 | -0.2 |
| **resting_**  **rmssd** |  |  |  |  |  | 1 | 0.55* |
| **oura_sleep_**  **score** |  |  |  |  |  |  | 1 |

N=15 days.

Table S2. Correlation between 2-weeks of daily assessment for the month of April

|  | **Positive Affect** | **Negative Affect** | **day_bpm** | **day_ rmssd** | **resting_bpm** | **resting_ rmssd** | **oura_sleep_score** |
| --- | --- | --- | --- | --- | --- | --- | --- |
| **Positive Affect** | 1 | 0.53* | 0.51 | -0.35 | 0.3 | -.28 | -0.43 |
| **Negative Affect** |  | 1 | 0.15 | -0.03 | 0.09 | 0.02 | -0.12 |
| **day_bpm** |  |  | 1 | -0.82*** | 0.87*** | -0.68** | -0.53 |
| **day_rmssd** |  |  |  | 1 | -0.9*** | 0.91*** | 0.08 |
| **resting_bpm** |  |  |  |  | 1 | -0.81*** | -0.28 |
| **resting_ rmssd** |  |  |  |  |  | 1 | 0.003 |
| **oura_sleep_ score** |  |  |  |  |  |  | 1 |

N=15 days.

Table S3. Correlation between 2-weeks of daily assessment for the month of June

|  | **Positive Affect** | **Negative Affect** | **day_bpm** | **day_ rmssd** | **resting_bpm** | **resting_ rmssd** | **oura_sleep_score** | **Step count** |
| --- | --- | --- | --- | --- | --- | --- | --- | --- |
| **Positive Affect** | 1 | 0.01 | -0.14 | -0.12 | 0.24 | -0.17 | -0.02 | 0.42 |
| **Negative Affect** |  | 1 | -0.5 | 0.65** | -0.47 | 0.14 | 0.49 | -0.13 |
| **day_bpm** |  |  | 1 | -0.83*** | 0.8*** | -0.46 | -0.51 | -.06 |
| **day_rmssd** |  |  |  | 1 | -0.68** | 0.44 | 0.4 | -.13 |
| **resting_bpm** |  |  |  |  | 1 | -0.49 | -0.53 | 0.12 |
| **resting_ rmssd** |  |  |  |  |  | 1 | 0.07 | 0.20 |
| **oura_sleep_ score** |  |  |  |  |  |  | 1 | -0.53 |
| **Step count** |  |  |  |  |  |  |  | 1 |

N=15 days; Step count for the month of September is included after IRB approval in April.

Table S4. Correlation between 2-weeks of daily assessment for the month of September

|  | **Positive Affect** | **Negative Affect** | **day_bpm** | **day_ rmssd** | **resting_bpm** | **resting_ rmssd** | **oura_sleep_score** | **Step count** |
| --- | --- | --- | --- | --- | --- | --- | --- | --- |
| **Positive Affect** | 1 | -0.57* | 0.19 | -0.09 | 0.43 | -0.46 | -0.63* | -0.35 |
| **Negative Affect** |  | 1 | -0.12 | -0.33 | 0.07 | -0.16 | 0.51 | -0.29 |
| **day_bpm** |  |  | 1 | -0.58* | 0.56* | -0.21 | -0.33 | 0.42 |
| **day_rmssd** |  |  |  | 1 | -0.54* | 0.31 | -0.07 | -0.08 |
| **resting_bpm** |  |  |  |  | 1 | -0.79*** | -0.21 | 0.58* |
| **resting_ rmssd** |  |  |  |  |  | 1 | 0.26 | -0.49 |
| **oura_sleep_ score** |  |  |  |  |  |  | 1 | -0.59* |
| **Step count** |  |  |  |  |  |  |  | 1 |

*N=15 days; we include Step count for the month of September because it was accessible.*
